# Tat-HSP22 inhibits oxidative stress-induced hippocampal neuronal cell death by regulation of the mitochondrial pathway

**DOI:** 10.1186/s13041-016-0281-8

**Published:** 2017-01-04

**Authors:** Hyo Sang Jo, Dae Won Kim, Min Jea Shin, Su Bin Cho, Jung Hwan Park, Chi Hern Lee, Eun Ji Yeo, Yeon Joo Choi, Hyeon Ji Yeo, Eun Jeong Sohn, Ora Son, Sung-Woo Cho, Duk-Soo Kim, Yeon Hee Yu, Keun Wook Lee, Jinseu Park, Won Sik Eum, Soo Young Choi

**Affiliations:** 1 Department of Biomedical Science and Research Institute of Bioscience and Biotechnology, Hallym University, Chuncheon, 24252 Korea; 2 Department of Biochemistry and Molecular Biology, Research Institute of Oral Sciences, College of Dentistry, Gangneung-Wonju National University, Gangneung, 25457 Korea; 3 Department of Biochemistry and Molecular Biology, University of Ulsan College of Medicine, Seoul, 05505 Korea; 4 Department of Anatomy, College of Medicine, Soonchunhyang University, Cheonan-Si, 31538 Korea

**Keywords:** Tat-HSP22, Oxidative stress, Apoptosis, Ischemia, Protein therapy

## Abstract

Oxidative stress plays an important role in the progression of various neuronal diseases including ischemia. Heat shock protein 22 (HSP22) is known to protect cells against oxidative stress. However, the protective effects and mechanisms of HSP22 in hippocampal neuronal cells under oxidative stress remain unknown. In this study, we determined whether HSP22 protects against hydrogen peroxide (H_2_O_2_)-induced oxidative stress in HT-22 using Tat-HSP22 fusion protein. We found that Tat-HSP22 transduced into HT-22 cells and that H_2_O_2_-induced cell death, oxidative stress, and DNA damage were significantly reduced by Tat-HSP22. In addition, Tat-HSP22 markedly inhibited H_2_O_2_-induced mitochondrial membrane potential, cytochrome *c* release, cleaved caspase-3, and Bax expression levels, while Bcl-2 expression levels were increased in HT-22 cells. Further, we showed that Tat-HSP22 transduced into animal brain and inhibited cleaved-caspase-3 expression levels as well as significantly inhibited hippocampal neuronal cell death in the CA1 region of animals in the ischemic animal model. In the present study, we demonstrated that transduced Tat-HSP22 attenuates oxidative stress-induced hippocampal neuronal cell death through the mitochondrial signaling pathway and plays a crucial role in inhibiting neuronal cell death, suggesting that Tat-HSP22 protein may be used to prevent oxidative stress-related brain diseases including ischemia.

## Introduction

Heat shock proteins (HSPs) are known to be stress-inducible proteins and are found in all organism. They play an important role in cell survival in cells exposed to biological stresses such as oxidative stress or chemical stress [1–3]. The HSP family are classified based on their molecular weight including HSP70, HSP90, HSP110. Small HSPs are highly conserved and well characterized as playing the role of molecular chaperones, and having a protective function against biological stress [4–7]. Among HSPs, HSP22 is known as a member of the family of small HSPs containing α-crystal domains, preferentially expressed in heart, and having protective effects against various conditions such as oxidative stress, aging, cancer, and apoptosis [8–11]. Other studies have demonstrated overexpression of HSP22 promotes cardioprotection in an ischemic preconditioning animal model by inhibition of iNOS production and mitochondrial dysfunction [12, 13].

The mitochondria play an important role in multiple cellular processes including apoptosis [14]. Mitochondria are known as one of the major sources of reactive oxygen species (ROS) production as a by-product of cellular processes. Excessive ROS causes damage to macromolecules, subsequently leading to mitochondria dysfunction and cell death [14–16]. Other studies have showed that mitochondrial dysfunction is associated with ROS production and mitochondria as well as with ischemic damage [12–16]. However, the exact biological function and precise mechanism of HSP22 protein in ischemic neuronal damage remains poorly understood.

Protein transduction domains (PTDs) facilitate the delivery of molecules including proteins into cells or tissues. Thus, PTDs were widely used as a tool to investigate the effect of target proteins in a number of diseases [17–20]. Many studies have demonstrated that PTD fusion protein transduced in vitro and in vivo and markedly inhibited stress induced cell death [21–26].

In this study, we cloned, expressed, and purified the Tat-HSP22 protein to examine the effect of Tat-HSP22 in ischemic neuronal damage. Our data demonstrate that Tat-HSP22 protein significantly inhibits oxidative stress-induced hippocampal HT-22 cell death and mitochondrial dysfunction, suggesting Tat-HSP22 protein may allow for the development of a therapeutic protein for neuronal diseases including ischemia.

## Results

### Identification of cell permeable Tat-HSP22 protein

**Fig. 1:**
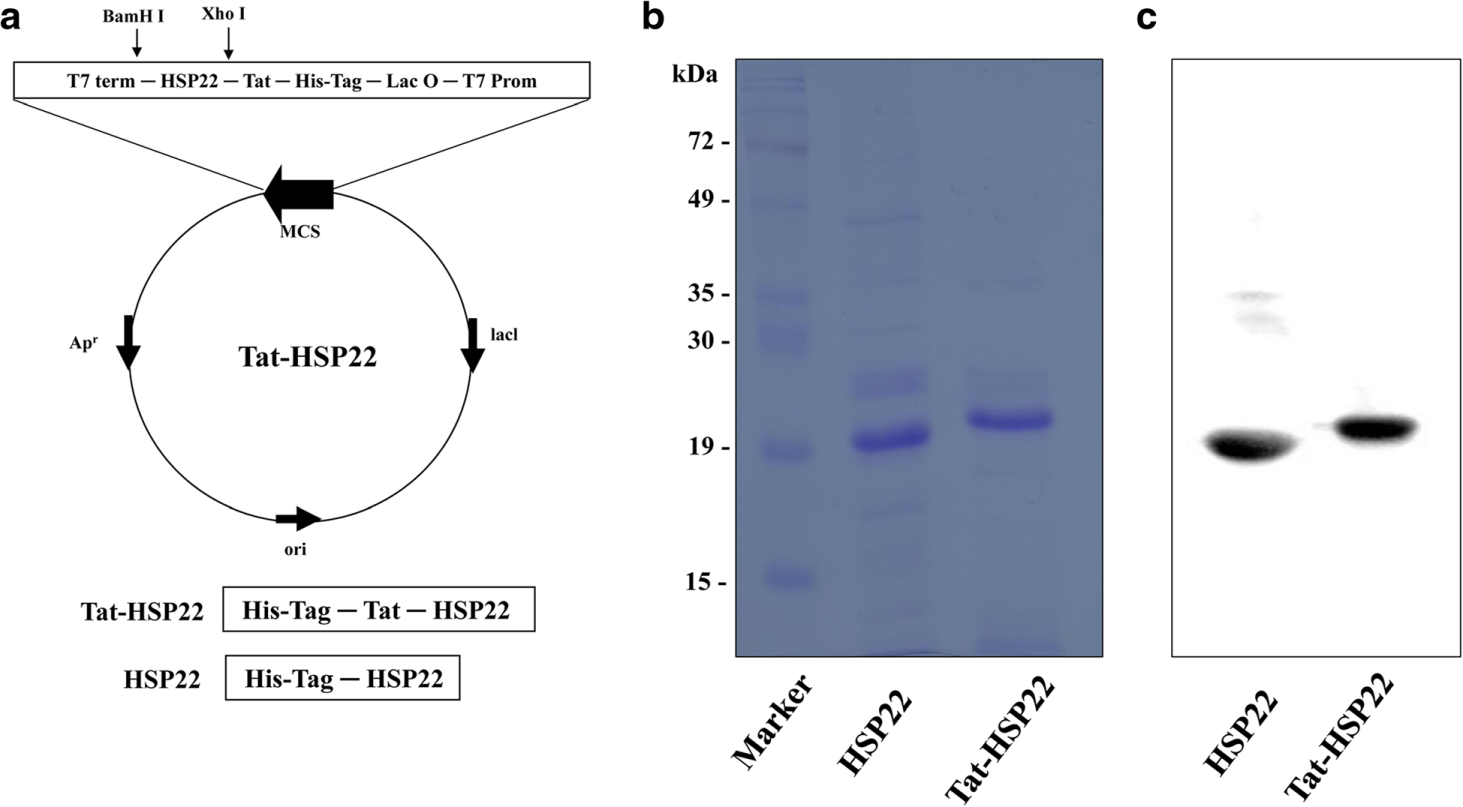
Construction and purification of Tat-HSP22 protein. **a** Diagram of expressed Tat-HSP22 and control HSP22 proteins. Tat-HSP22 expression vector was constructed using pET15b vector. Purification of Tat-HSP22 and control HSP22 protein. After proteins were expressed by IPTG, the proteins were purified using Ni-NTA affinity chromatography. Then, purified proteins were analyzed by **b** 12% SDS-PAGE and **c** Western blot analysis with an anti-histidine antibody

The full length human HSP22 gene was obtained from a liver cDNA library using PCR. The produced gene was digested with restriction enzymes (*Xho* I and *Bam* HI) and the gene was cloned into a Tat expression vector to produce cell permeable Tat-HSP22 protein. As shown in Fig. 1a, the resulting Tat-HSP22 protein expression vector contained histidine residue and Tat peptide whereas a HSP22 expression vector was constructed as a control without the Tat peptide. The constructed Tat-HSP22 and control HSP22 protein expression vectors were confirmed by digestion with the same restriction enzymes (*Xho* I and *Bam* HI) and DNA sequencing analysis (data not shown).

To purify the Tat-HSP22 and control HSP22 protein, the proteins were induced by adding 0.5 mM IPTG and cultured at 37 °C for 4 h. After expression of Tat-HSP22 and control HSP22 proteins, these proteins were purified using Ni-NTA affinity chromatography. Subsequently, we performed PD-10 column chromatography to removed salt from the purified proteins. SDS-PAGE and Western blot analysis using an anti-histidine antibody showed that purified Tat-HSP22 and control HSP22 protein bands correspond with the expected molecular weight of HSP22 protein (Fig. 1b and c).

### Transduction ability of Tat-HSP22 protein into HT-22 cells

**Fig. 2:**
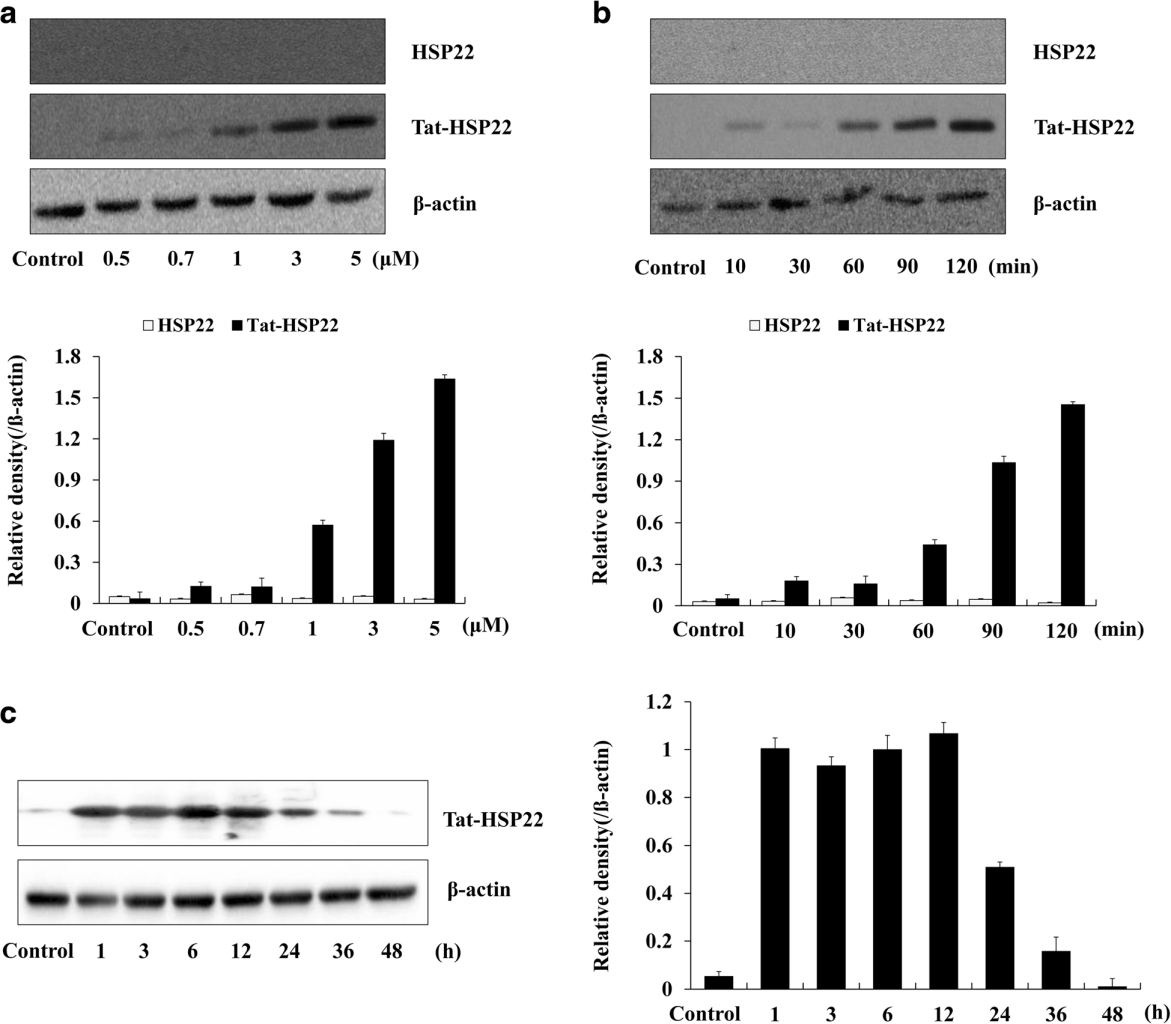
Transduction efficiency of Tat-HSP22 protein into HT-22 cells. **a** Tat-HSP22 and control HSP22 proteins (0.5–5 μM) were added to the culture media for 2 h, **b** Tat-HSP22 and control HSP22 proteins (5 μM) were added to the culture media for 10–120 min and analyzed by Western blot and the band intensities were measured by densitometer. **c** Intracellular stability of Tat-HSP22 protein. The cells pretreated with 5 μM Tat-HSP22 protein were incubated for 1–48 h and analyzed by Western blot and the band intensities were measured by densitometer

To determine whether Tat-HSP22 proteins transduced into hippocampal HT-22 neuronal cells, HT-22 was treated with Tat-HSP22 or control HSP22 proteins (0.5-5 μM) for 2 h or with Tat-HSP22 or control HSP22 proteins (5 μM) over various time periods (10–120 min). The levels of transduced Tat-HSP22 protein were measured by Western blotting using an anti-histidine antibody. Westering blot analysis indicated that Tat-HSP22 protein transduced into HT-22 cells concentration- and time-dependently, whereas the control HSP22 protein was not detected in the cells (Fig. 2a and b).

**Fig. 3:**
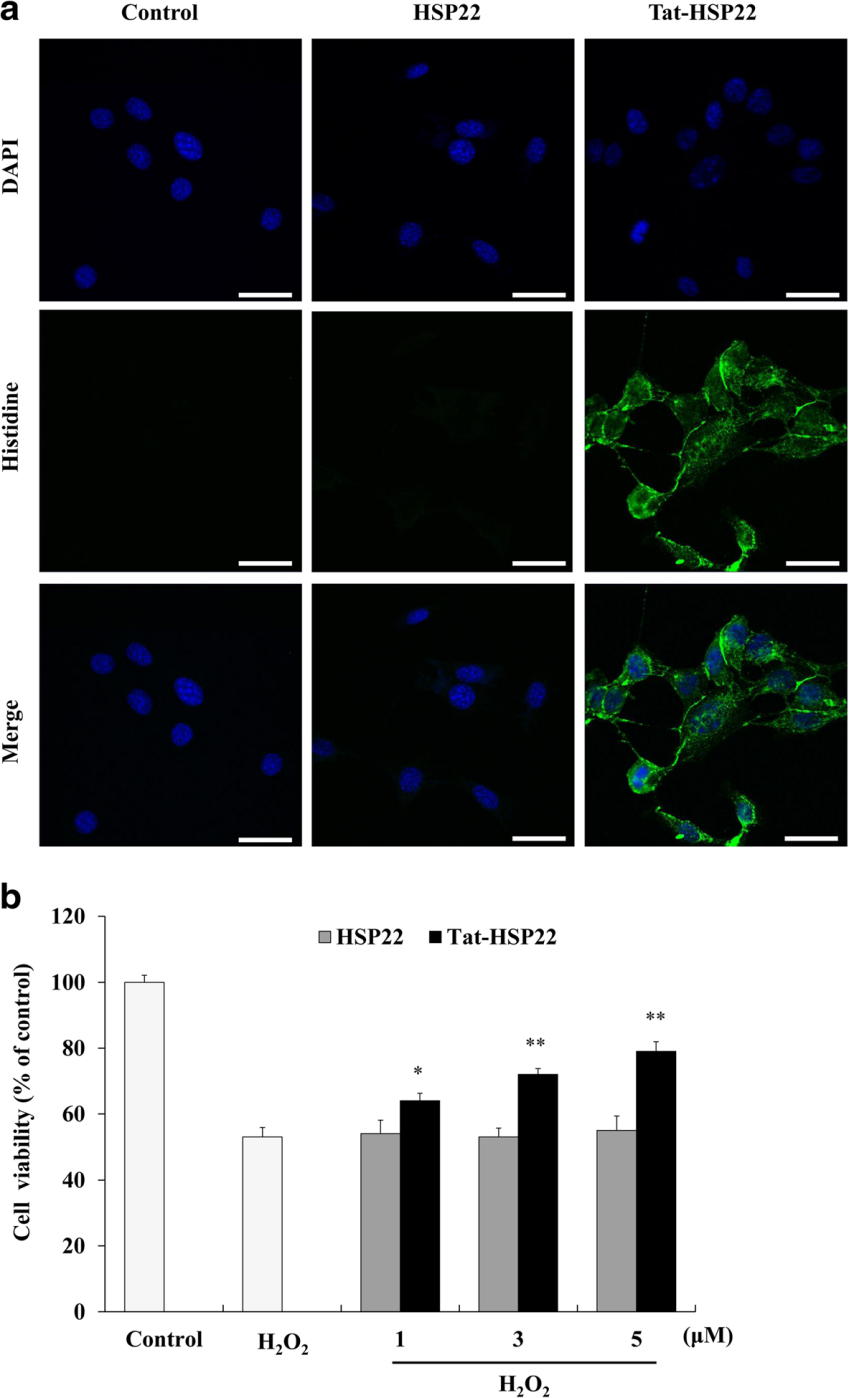
Transduced Tat-HSP22 protein inhibits H_2_O_2_-induced HT-22 cell death. **a** Cellular distribution of transduced Tat-HSP22 protein was analyzed using confocal microscopy. HT-22 cells were pretreated with 5 μM of Tat-HSP22 or control HSP22 protein for 2 h and the cells were examined by Histidine and DAPI staining. Scale bar = 20 μm. **b** The cells were pretreated with Tat-HSP22 or control HSP22 protein (5 μM) for 2 h and the cells were incubated in the absence or presence of H_2_O_2_ (1 mM) for 12 h. Cell viability was determined using WST-1 assay. ^**^ *P* < 0.01 compared with H_2_O_2_-treated cells

To evaluate how long transduced Tat-HSP22 protein is stable in HT-22, HT-22 was exposed to Tat-HSP22 protein for 2 h and the cells were cultured for various times to assess the stability of Tat-HSP22 protein. Tat-HSP22 protein stability was determined by Western blot analysis using an anti-histidine antibody. Significant levels of transduced Tat-HSP22 proteins persisted for 36 h in HT-22 cells (Fig. 2c). We also examined transduced Tat-HSP22 distribution using fluorescence microscopy analysis. As shown in Fig. 3a, the green fluorescence signals were not evident in the control HSP22 protein treated cells. However, more green fluorescence signals were evident in the cytosol and nucleus of cells treated with Tat-HSP22 protein than was evident in the control HSP22 protein treated cells, indicating that Tat-HSP22 protein transduce into HT-22.

### Tat-HSP22 protein inhibits against H_2_O_2_-induced HT-22 cell death

To investigate the effects of Tat-HSP22 protein against HT-22 cell death, a WST-1 assay was performed. HT-22 cells were treated with different doses of Tat-HSP22 protein (1–5 μM) for 2 h in the presence or absence of H_2_O_2_ (1 mM) for 12 h. The cell viability of HT-22 cells is shown in Fig. 3b. Our results showed that transduced Tat-HSP22 protein markedly increased cell viability from 53 to 79% in the H_2_O_2_ treated cells. In contrast, control HSP22 protein showed no protective effect against H_2_O_2_ induced cell death.

**Fig. 4:**
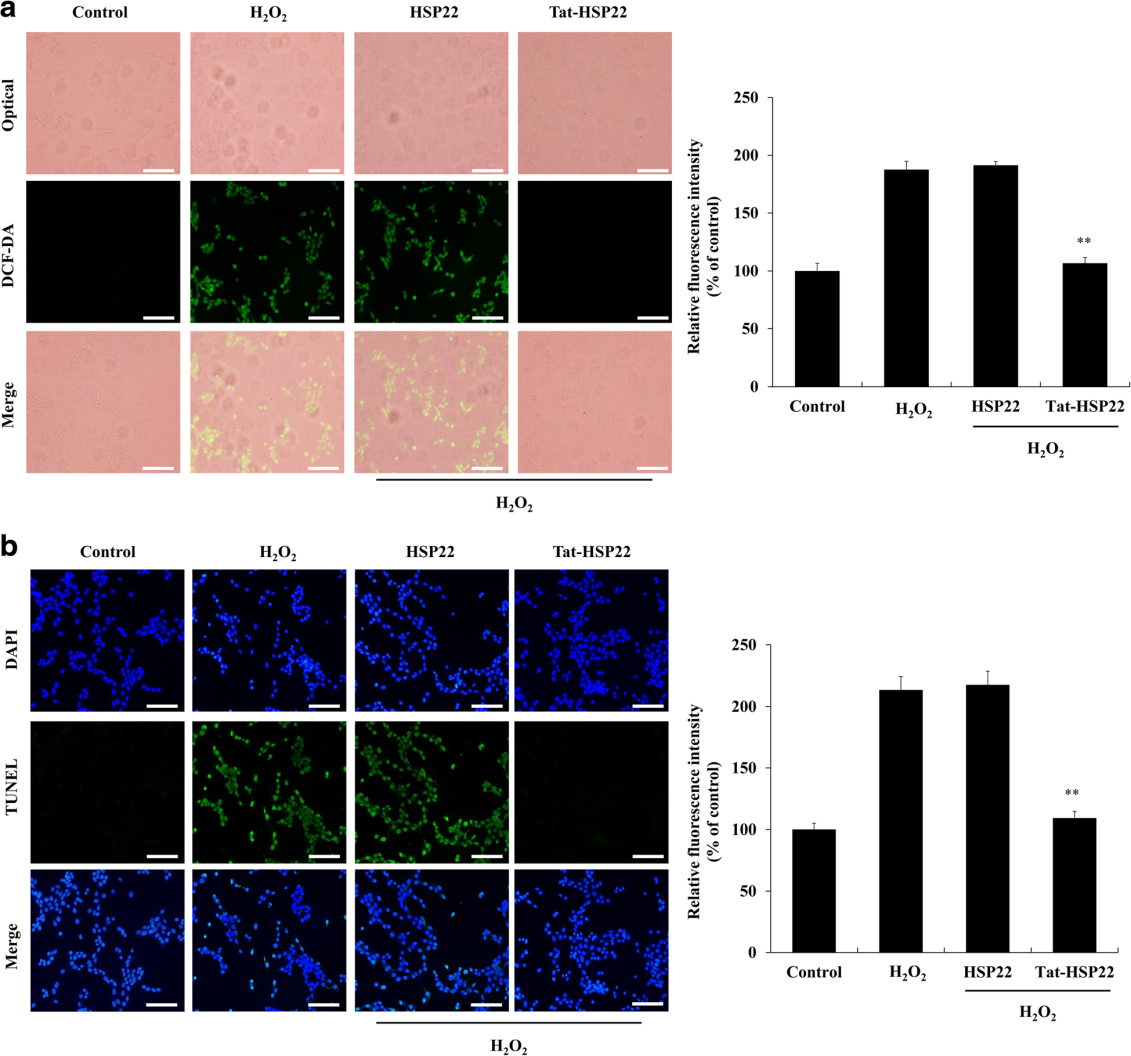
The effect of Tat-HSP22 protein against H_2_O_2_-induced intracellular toxicities in HT-22 cells. HT-22 cells were pretreated with 5 μM of Tat-HSP22 or control HSP22 protein for 2 h and the cells were incubated in the absence or presence of H_2_O_2_ (1 mM) for 10 min and 6 h, respectively. Then, **a** H_2_O_2_-induced oxidative stress levels were measured using DCF-DA staining and the fluorescence intensity was measured using an ELISA plate reader. **b** DNA fragmentation was detected by TUNEL staining and quantitative evaluation of TUNEL positive cells confirmed by cell counting under a phase-contrast microscopy. Scale bar = 50 μm. ^**^ *P* < 0.01 compared with H_2_O_2_-treated cells

Further, we examined the effects of Tat-HSP22 protein on the production of the intracellular ROS and levels of DNA damage in H_2_O_2_ exposed HT-22 cells by fluorescence staining. The cells were pretreated with Tat-HSP22 protein (5 μM) for 2 h and the cells were exposed to 1 mM H_2_O_2_ for 2 h. Then, the intracellular ROS and DNA damage were determined by DCF-DA and TUNEL staining (Fig. 4). We clearly observed that H_2_O_2_ exposure leads to markedly increased production of intracellular ROS and DNA damage, while cells pretreated with Tat-HSP22 protein showed drastically reduced intracellular ROS and DNA damage levels compared with H_2_O_2_ exposed HT-22 cells. In contrast, the results of control HSP22 protein showed no inhibitory effect compared with H_2_O_2_ exposed HT-22 cells, suggesting the effects of Tat-HSP22 against oxidative stress were caused by inhibiting H_2_O_2_-induced intracellular toxicities.

### Tat-HSP22 protein inhibits against H_2_O_2_-induced mitochondrial dysfunction and apoptosis

**Fig. 5:**
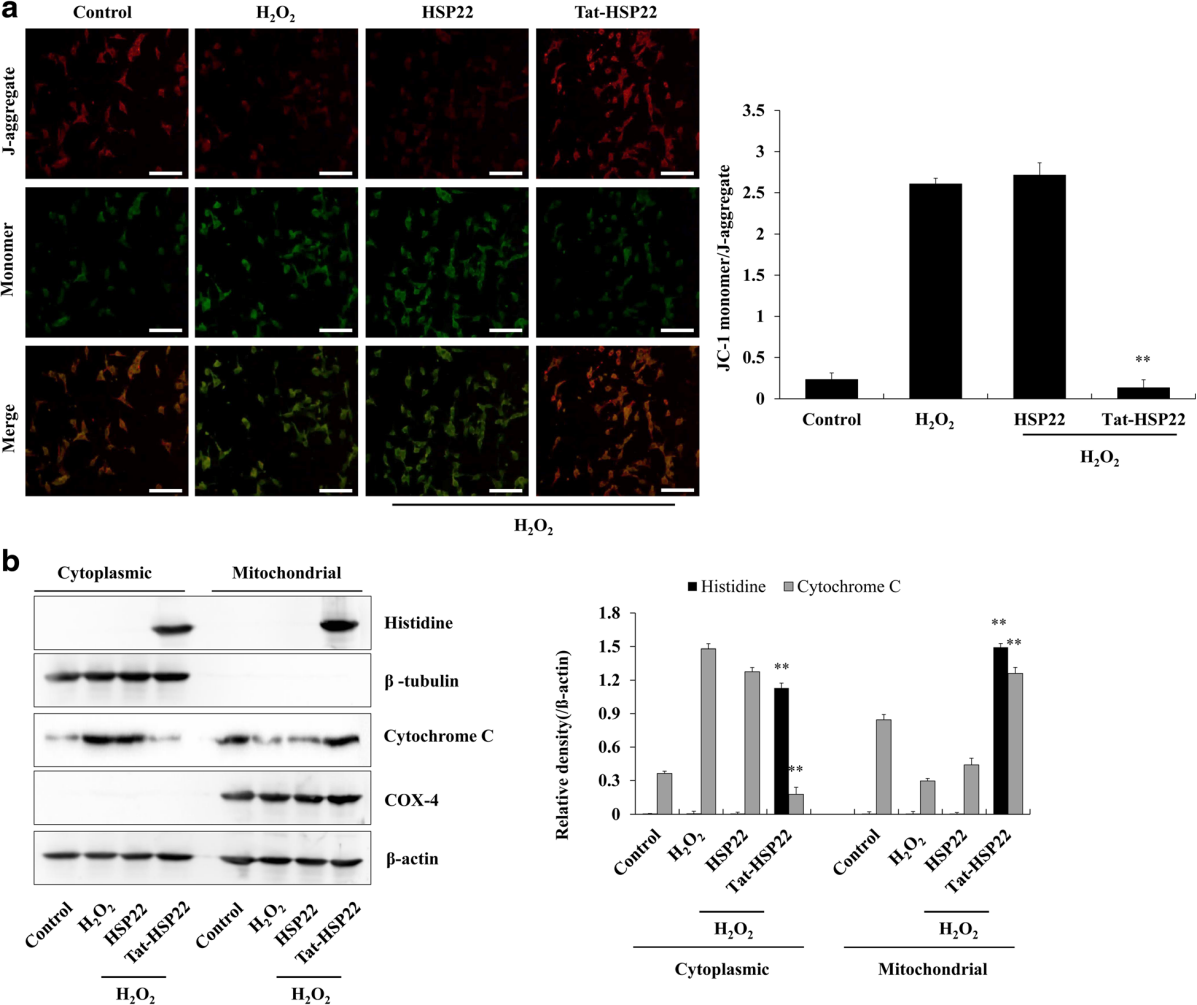
Tat-HSP22 protein protects against changes to H_2_O_2_-induced mitochondrial membrane potential and cytochrome *c* release. HT-22 cells were pretreated with 5 μM of Tat-HSP22 or control HSP22 protein for 2 h and exposed to H_2_O_2_ (1 mM) for 20 min and 4 h, respectively. **a** Changes of mitochondrial membrane potential were measured using JC-1 assay kit. Scale bar = 50 μm. **b** After mitochondria and cytosol fraction were separated using mitochondria isolation kit, transduced Tat-HSP22 protein and cytochrome *c* levels were determined using Western blotting in each fraction and band intensity by densitometer. ^**^ *P* < 0.01 compared with H_2_O_2_-treated cells

To elucidate the effects of Tat-HSP22 protein on mitochondrial dysfunction, we assessed changes in the mitochondrial membrane potential using JC-1 staining (Fig. 5a). The results showed that when HT-22 cells were exposed to H_2_O_2_, mitochondrial membrane potential was significantly increased compared to the normal control or control HSP22 protein treated cells. However, the increased levels of mitochondrial membrane potential in the H_2_O_2_ exposed cells were significantly inhibited by Tat-HSP22 protein.

Next, we determined the inhibitory effects of Tat-HSP22 protein on the suppression of H_2_O_2_-induced apoptosis. First, we determined the effect of Tat-HSP22 on transduction into mitochondria of HT-22 cells and changes of cytochrome *c* expression levels in separated cytosol and mitochondria fractions (Fig. 5b). We observed that Tat-HSP22 protein increased mitochondria fraction compared to the control cells. In addition, Tat-HSP22 protein markedly inhibited cytochrome *c* levels in the mitochondria fraction induced by H_2_O_2_. In comparison, control HSP22 protein did not change the cytochrome *c* levels. The results indicate that transduced Tat-HSP22 protein significantly inhibited cytochrome *c* release from mitochondria to cytosol induced by oxidative stress, suggesting Tat-HSP22 protein suppresses apoptosis induced by oxidative stress.

**Fig. 6:**
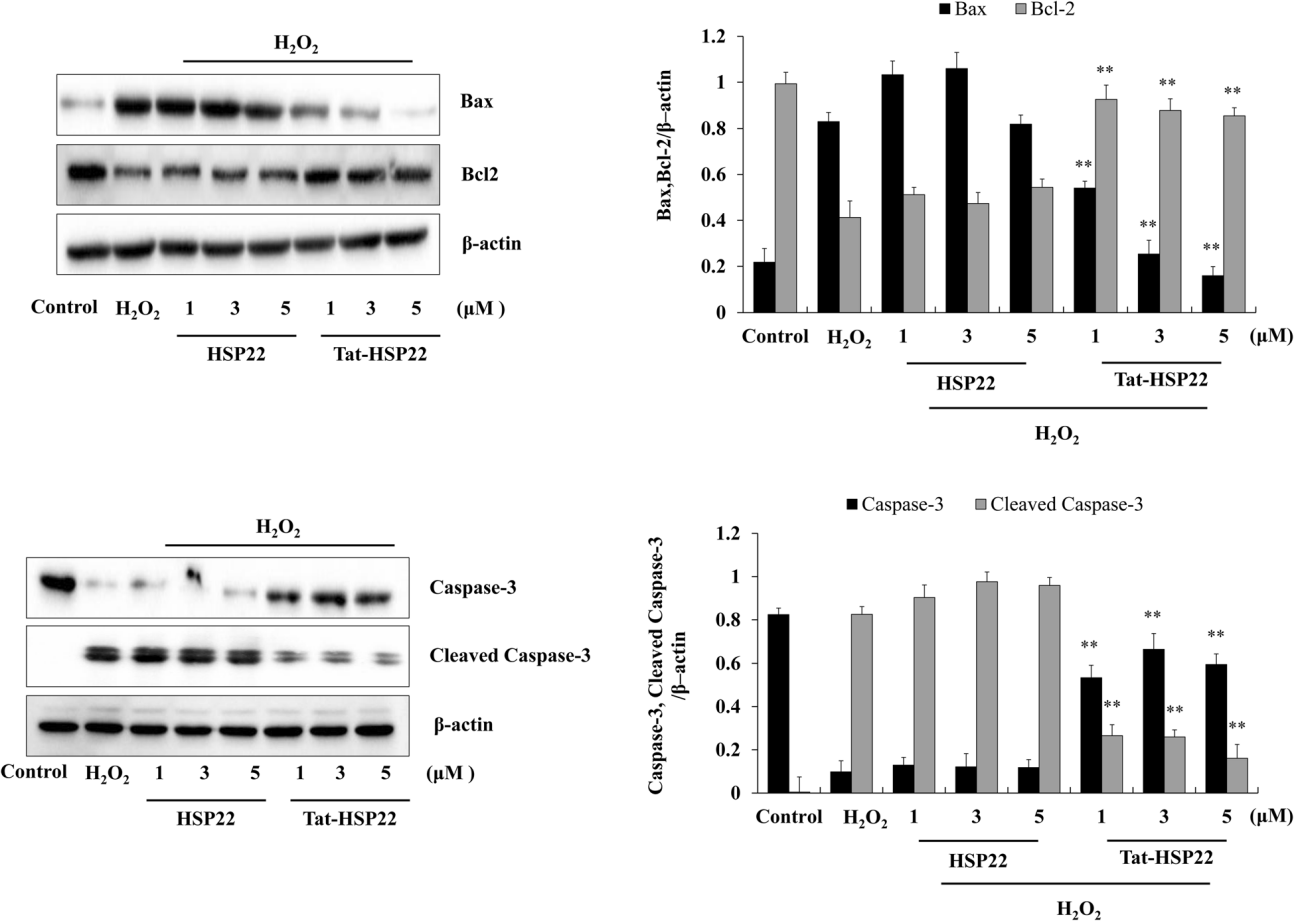
Effect of Tat-HSP22 protein against H_2_O_2_-induced Bax, Bcl-2, and caspase-3 expression levels in HT-22 cells. The cells were pretreated with 5 μM of Tat-HSP22 or control HSP22 protein for 2 h and exposed to H_2_O_2_ (1 mM) for 4 h and 6 h, respectively. Then, the levels of Bax, Bcl-2, caspase-3 and cleaved caspase-3 were measured by Western blotting and band intensity by densitometer. ^**^ *P* < 0.01 compared with H_2_O_2_-treated cells

Since apoptosis related proteins (Bax, Bcl-2, caspase-3, and cleaved caspase-3) levels were known to change during apoptosis, we determined the expression levels of these proteins (Fig. 6). Tat-HSP22 protein markedly reduced the expression of Bax and cleaved caspase-3 levels compared to the cells exposed to H_2_O_2_ alone, while Bcl-2 and caspase-3 expression levels were significantly increased compared to the H_2_O_2_ exposed cells. However, these protein levels were did not change in control HSP22 protein treated cells under the same experimental condition. These results suggest that the Tat-HSP22 protein protects against oxidative stress-induced apoptosis by regulation of apoptosis related protein expression levels.

### Neuroprotective effects of Tat-HSP22 protein in CA1 region after ischemic damage

**Fig. 7:**
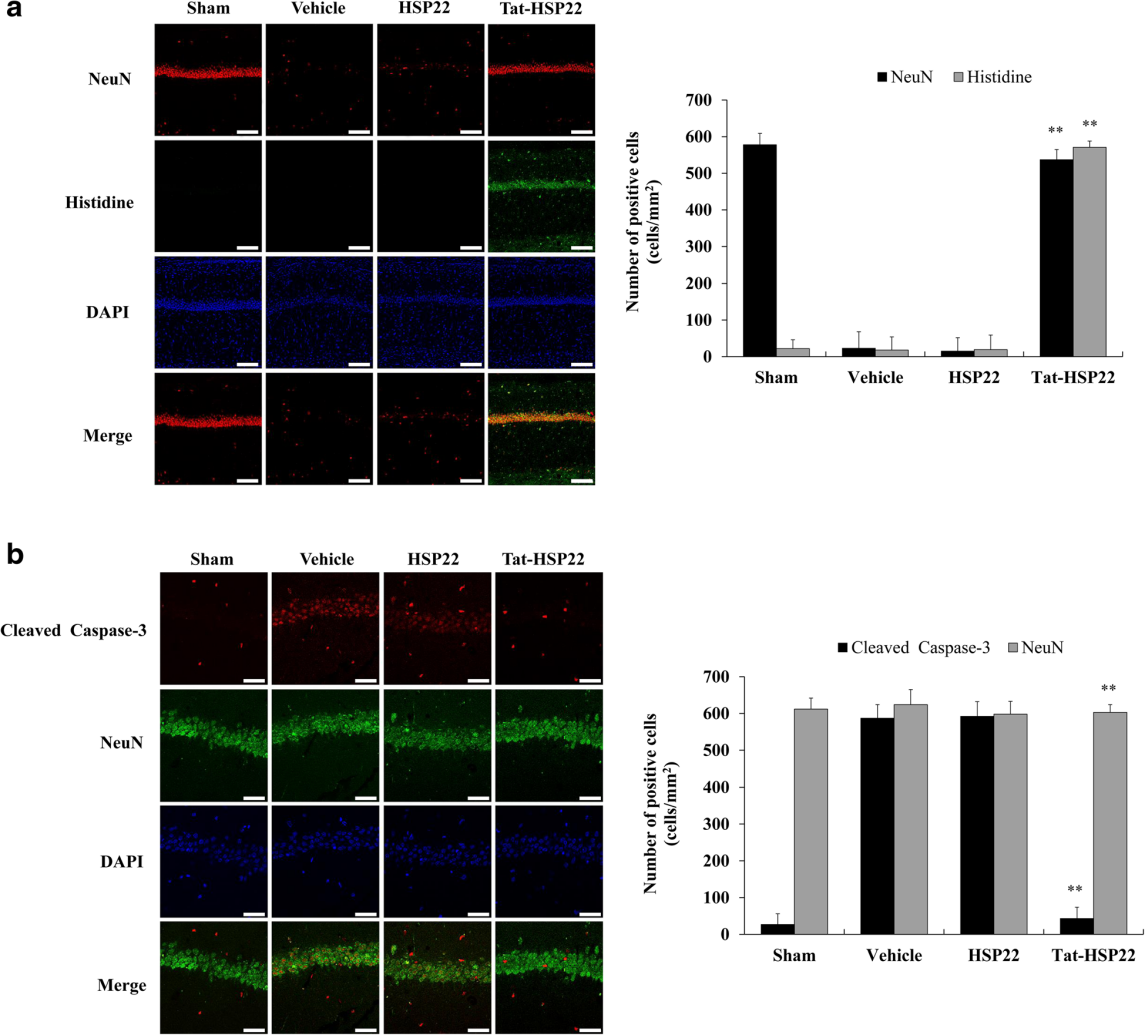
Transduction of Tat-HSP22 protein into gerbil brain and its protective effects. Gerbils were treated with single injections of Tat-HSP22 (2 mg/kg) or control HSP22 protein and killed. **a** 7 days after ischemic insults, transduced Tat-HSP22 protein was confirmed using an anti-Histidine, NeuN, DAPI, and Alexa immunostaining. Scale bar = 50 μm. **b** The changes of cleaved caspase-3 expression levels were confirmed using an anti-cleaved caspase-3 staining after 12 h ischemic insults. Scale bar = 18.8 μm. ^**^ *P* < 0.01, significantly different from the vehicle group

We determined the effects of transduced Tat-HSP22 protein on animal brain tissue and its ability to cross the BBB. Because the BBB prevents the delivery of therapeutic agents into the brain, crossing the BBB is a key point to protect ischemic injury. To confirm that Tat-HSP22 proteins transduce into the hippocampal CA1 region and protect against ischemic injury, we performed Histidine and NeuN immunostaining. As shown in Fig. 7a, Tat-HSP22 protein transduced into the hippocampal CA1 region of animal brain and markedly protected neurons damage induced by ischemic injury.

We also determined whether cleaved caspase-3 expression was involved in ischemic injury. To confirm the protective effect of Tat-HSP22 protein against ischemic injury derived cleaved caspase-3 expression, cleaved caspase-3 and NeuN immunostaining were performed 12 h after ischemia-reperfusion (Fig. 7b). Immunostaining showed that control HSP22 protein did not protect against ischemic injury. Tat-HSP22 protein significantly prevents the cleaved caspase-3 expression levels induced by ischemic injury, indicating that Tat-HSP22 protein has the ability to transduce into animal brain and protect neurons in the face of ischemic injury.

**Fig. 8:**
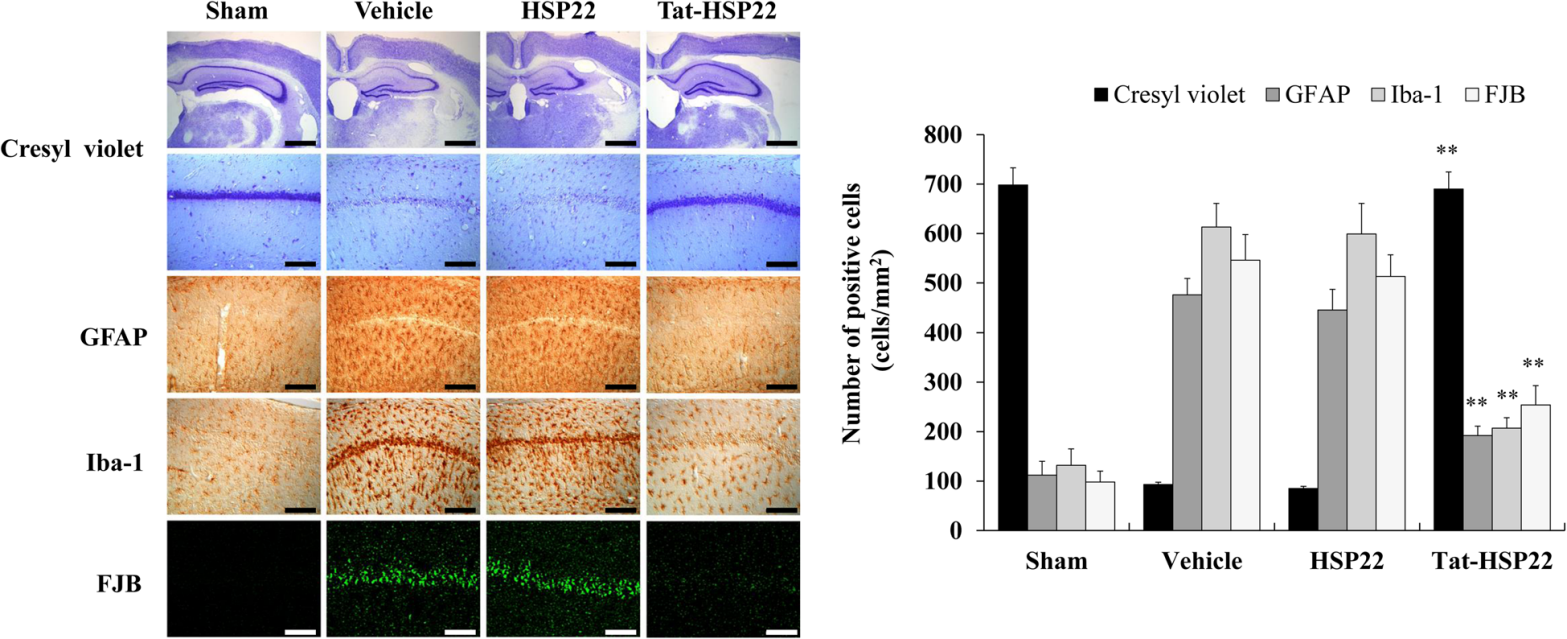
Neuroprotective effects of Tat-HSP22 protein against ischemic damage. Gerbils were treated with single injections Tat-HSP22 (2 mg/kg) or control HSP22 protein and killed after 7 days. Neuronal cell viability after ischemic insults were determined using Cresyl violet, GFAP, Iba-1, and F-JB immunostaining. Relative numeric analysis of CV, GFAP, Iba-1, and F-JB positive neurons in the CA1 region. Scale bar = 400 and 50 μm. ^**^ *P* < 0.01, significantly different from the vehicle group

Further, we examined the protective effects of Tat-HSP22 protein against ischemic injury (Fig. 8). Cresyl violet (CV) immunohistochemistry showed that Tat-HSP22 protein markedly increased neuronal cell viability in ischemic injury compared to the vehicle group. In addition, we confirmed neuronal degeneration in the CA1 region using Fluoro-Jade B (F-JB) staining since neuronal degeneration causes neuronal cell death. Tat-HSP22 protein markedly protects against neuronal degeneration induced by ischemic injury. In contrast, control HSP22 protein showed the similar results as the vehicle group. Our results demonstrate that Tat-HSP22 protein efficiently promotes neuronal cell survival induced in ischemic injury.

Since the activation of astrocytes and microglia are known to be strongly involved in ischemic injury, we further performed GFAP and Iba-1 staining to detect whether Tat-HSP22 protein prevents astrocytes and microglia activation induced by ischemic injury. GFAP and Iba-1 staining showed that control HSP22 protein demonstrated a similar pattern as the vehicle group, while Tat-HSP22 protein drastically protected against astrocytes and microglia activation induced by ischemic injury. As shown in Fig. 8, our results indicate that Tat-HSP22 protein significantly prevented the neuronal cell death in the CA1 region induced by ischemic injury.

## Discussion

Heat shock protein 22 (HSP22) known as H11 kinase, E21G1, and HSPB8 is a member of the small HSP family of proteins containing a α-crystalline domain and has been demonstrated to have protective effect against various conditions including oxidative stress, aging, cancer, and apoptosis [8–13]. Although HSP22 protein is well known to function in the pathophysiological processes of various cancer cells, the exact function and mechanism of HSP22 protein in hippocampal neuronal cell damage from oxidative stress and in an ischemic brain injury animal model remains to be clarified. Therefore, we investigated the effects of HSP22 protein against oxidative stress-induced neuronal cell damage to clarify the function and mechanism used Tat-HSP22 protein, which is known for the ability to be efficiently delivered into cells or tissues. Since HIV-1 Tat protein transduction domain (PTD) fusion protein has the ability to efficiently transduce into cells by crossing the plasma membrane or brain tissue therefore crossing the blood-brain barrier (BBB), numerous studies have demonstrated the potential of its therapeutic protein application in cells or tissues against various diseases [11, 17–26].

In this study, we constructed the recombinant Tat-HSP22 protein expression vector and purified Tat-HSP22 protein with Ni-NTA affinity chromatography. Purified Tat-HSP22 protein was evaluated by SDS-PAGE and Western blot analysis. We subsequently confirmed the transduction ability of Tat-HSP22 protein into HT-22 cells. Our data showed that Tat-HSP22 protein transduced into HT-22 cells dose- and time-dependently. In addition, we confirmed the distribution of transduced Tat-HSP22 protein in HT-22 cells by immunofluorescence staining. Transduced Tat-HSP22 protein distributed in the cytosol and nuclei of HT-22 cells. These results indicate that we successfully constructed Tat-HSP22 protein expression vector and purified Tat-HSP22 protein efficiently transduced into HT-22 cells. Consistent with other studies, which have demonstrated that various purified PTD fusion proteins efficiently transduce into cells [19–25, 27, 28].

Hydrogen peroxide (H_2_O_2_), a mild oxidant, is a known by-product of a normal biological processes in cells. However, high levels of H_2_O_2_ are very toxic and critical for cell survival and signaling pathways because reactive oxygen species (ROS) including superoxide anion and hydroxyl radical are produced in the presence of high levels of H_2_O_2_ [29–31]. Also, generated ROS is well known to contribute to cellular damage by destroying macromolecules including proteins, lipids, and nucleotides [32]. Thus, we examined the effect of Tat-HSP22 protein against H_2_O_2_-induced cellular toxicities. We demonstrated that transduced Tat-HSP22 protein significantly increased cell survival compared to the cells treated only with H_2_O_2_-treated. Also, Tat-HSP22 protein markedly inhibited H_2_O_2_-induced intracellular oxidative stress and DNA fragmentation levels compared to the H_2_O_2_-treated cells. Other studies have shown that overexpression of HSP22 promotes cardiac cell survival and HSP22 has protective effects in the stress condition of cardiac cells via regulating the PI3K/Akt pathways [33]. Also, overexpression of HSP22 increased resistance to oxidative stress and longevity in flies whereas the absence of HSP22 expression decreased the lifespan and resistance to stress, suggesting that HSP22 overexpression play beneficial roles during aging [34, 35]. In agreement with these results, we have shown that transduced Tat-HSP22 protein prevents HT-22 cell death caused by H_2_O_2_-induced oxidative stress.

Several studies have demonstrated that mitochondria is the main generator of ROS and excessive ROS eventually leads to mitochondria dysfunction and cell death by induction of apoptosis in various conditions including aging and ischemic injury [36–38]. Further, apoptosis is known to play a critical role in the survival of cells in various conditions including ischemia. The balance of pro-apoptotic (Bax) and anti-apoptotic (Bcl-2) proteins is important to cell survival. Activation of Bax induced cell death by cytochrome *c* release from the mitochondria and activation of caspase-3 [39, 40]. Thus, we determined the effect of transduced Tat-HSP22 protein on H_2_O_2_-induced mitochondria apoptotic signal pathway. Tat-HSP22 protein significantly prevented mitochondrial dysfunction and inhibited the release of cytochrome *c* from mitochondria to cytosol as well as inhibited cell death via regulation of the expression of the apoptotic proteins Bax, Bcl-2, and active caspase-3. Corroborating our results, other studies have demonstrated that overexpression of HSP22 protein increase resistance to oxidative stress and prevents mitochondrial dysfunction by reducing ROS production during the aging process of flies [14]. Also, overexpression of HSP22 protein inhibits the mitochondria dependent apoptosis through regulation of pro-apoptotic and anti-apoptotic proteins expression in myocardial infarction [41].

Since it is difficult to cross the BBB it is difficult to deliver therapeutic drugs or agents into brain, the therapeutic effects of drugs or agents is limited in neuronal diseases including ischemia. However, PTD fusion proteins are known to be able to delivery therapeutic proteins into the brain which is considered a powerful tool in the development of therapeutic drugs or agents in neuronal diseases [17–19, 42]. In a previous study, we demonstrated that Tat fused protein inhibited neuronal cell death in ischemic injury via crossing the BBB [22]. In this study, we determined the effect of Tat-HSP22 protein against ischemia in an animal model. Our results showed that Tat-HSP22 protein efficiently transduced into the hippocampal CA1 region where it markedly inhibited neuronal cell death. Tat-HSP22 protein also decreased active caspase-3 expression levels in the hippocampal CA1 region. Several studies have shown that HSP22 protein expression is increased in a swine ischemia model and in ischemia patients, suggesting that HSP22 protein plays protective roles in ischemic injury [43, 44]. In addition, HSP22 protein enhances cardiac cell survival by prevention of myocardial infarction in ischemic preconditioning [41]. Other studies have also shown that increased HSP22 mRNA is associated with the improvement in renal function after ischemia [45].

Several studies have demonstrated that the activation of microglia and astrocytes as well as the expression of abundant glial fibrillary acidic protein are highly associated with neuronal diseases including ischemic injury [46–49]. Using GFAP, Iba-1, and F-JB immunohistochemistry, we demonstrated that Tat-HSP22 protein markedly reduced activation of microglia and astrocytes as well as the expression of glial fibrillary acidic protein in the hippocampal CA1 region. These results indicate that Tat-HSP22 protein plays an important role in preventing neuronal damage via regulation of gliosis and neuronal degeneration resulting from ischemia. However, further studies are required to understand the exact mechanisms in ischemic injury.

In conclusion, we showed that Tat-HSP22 protein transduced into hippocampal HT-22 cells in vitro and the CA1 region in vivo and significantly inhibited neuronal cell death caused by oxidative stress, suggesting that Tat-HSP22 protein provides a potential therapeutic strategy to prevent oxidative stress-induced diseases including ischemic injury.

## Methods

### Cell culture and cell viability assay

Hippocampal neuronal cells (HT-22) were cultured as described previously [27]. To assess cell viability, HT-22 cells were preincubated with Tat-HSP22 or control HSP22 (1–5 μM) for 2 h before treatment with hydrogen peroxide (1 mM) for 12 h. Cell viability was determined by a WST-1 kit (Daeillab Service, Seoul, Korea) [27].

### Construction, purification, and transduction of recombinant Tat-HSP22 into HT-22

The HSP22 full length cDNA amplification was performed using human HSP22 specific primers as follows; the primer of forward as 5′-CTCGAGATGGCTGACGGTCAG-3′ and the primer of reverse as 5′-GGATCCTCAGGTACAGGTGACTTCCT-3′. Subsequently, we constructed cell permeable Tat-HSP22 and control HSP22 expression vector as previously described [22].

After transformation of constructed Tat-HSP22 and control HSP22 protein expression vector into *Escherichia coli* BL21 (DE3), these plasmids were cultured in LB medium at 37 °C for 4 h including ampicillin (50 μg/ml) and IPTG (0.5 mM) to purify Tat-HSP22 and control HSP22 protein. Then, overexpressed Tat-HSP22 and control HSP22 proteins were harvested by centrifugation and the proteins were purified using affinity chromatograph as described in a previous study [22]. Purified Tat-HSP22 protein concentration was determined using a Bradford assay [50].

For transduction of Tat-HSP22 into HT-22, the proteins were added to the HT-22 culture medium at different concentrations (0.5–5 μM) for 2 h or treated over various times (10–120 min) at the same level (5 μM). Then, the HT-22 was washed with trypsin-EDTA and PBS. The levels of transduced Tat-HSP22 was identified by Western blotting and confocal microscope analysis (FV-300; Olympus, Tokyo, Japan) [23]. Western blotting was performed as previously described [23, 51]. The protein bands were analyzed ECL (Amersham, Franklin Lakes, NJ, USA) using the indicated specific antibody.

### Fluorescence microscopy analysis

To detect the intracellular distribution of transduced Tat-HSP22 into HT-22, fluorescence microscopy was performed as described previously [24]. After exposure of HT-22 to Tat-HSP22 (5 μM) for 2 h, the cells were washed twice with PBS. Subsequently, the cells were staining with Histidine primary Ab and Alexa Fluor 488-conjugated secondary Ab. After washing, the cells were staining with DAPI (Roche, Mannheim, Germany) and observed under a FV-300 confocal microscope (Olympus, Tokyo, Japan).

### Measurement of intracellular toxicities

To examine the function of Tat-HSP22 against oxidative stress-induced HT-22 cell toxicities, HT-22 were treated with Tat-HSP22 (5 μM) after exposure or non-exposure to hydrogen peroxide for 2 h. Then, the hydrogen peroxide-induced cellular toxicities including cellular ROS (DCF-DA staining), DNA fragmentation (TUNEL staining), and mitochondria dysfunction levels (JC-1 staining) were measured by fluorescence microscopy analysis [22–24, 52].

### Experimental animals

Male gerbils obtained from the Experimental Animal Center, at Hallym University were housed at 23 °C, with humidity of 60%, and exposed to 12 h periods of light and dark with free access to food and water. All experimental procedures involving animals and their care conformed to the Guide for the Care and Use of Laboratory Animals of the National Veterinary Research & Quarantine Service of Korea and were approved by the Institutional Animal Care and Use Committee of Soonchunhyang University [SCH 15-0005].

### Effects of Tat-HSP22 on ischemic animal model

To investigate the effects of HSP22 against ischemic insults, the animals were divided into 4 groups (7 gerbils in each groups). Group 1, sham operated control group; group 2, ischemia operated vehicle group; group 3, control HSP22 protein (2 mg/kg) + ischemia operated group; group 4, Tat-HSP22 protein (2 mg/kg) + ischemia operated group. Control HSP22 and Tat-HSP22 proteins were intraperitoneally injected 30 min after ischemia-reperfusion.

In addition, we examined changes in cleaved caspase-3 expression levels caused by Tat-HSP22 protein in an ischemic animal model. 30 min after ischemia-reperfusion, control HSP22 and Tat-HSP22 proteins were intraperitoneally injected, the animals were sacrificed after 12 h to confirm the changes of cleaved caspase-3 expression levels by immunostaining.

Induction of ischemic injury was performed as previously described [22, 27]. Briefly, ischemia was induced by the occlusion of arteries with non-traumatic aneurysm clips. 7 days after induction of ischemic injury, the effects of HSP22 proteins were examined by immunohistochemistry [22, 27, 28].

### Immunostaining and immunohistochemistry analysis

To determine the effects of transduced Tat-HSP22 protein against neuronal cell death and changes of cleaved-capase-3 expression levels in ischemic animal model, NeuN, histidine, and cleaved caspase-3 immunostaining were performed. Briefly, the sections were stained with NeuN (diluted 1:1,000; Chemicon International, Temecula, CA, USA), histidine (diluted 1:1,000; Santa Cruz, CA, USA), cleaved caspase-3 (diluted 1:1,000; Cell Signaling Technology, Beverly, MA, USA). The sections were also incubated in a mixture of FITC- and Cy3-conjugated secondary antisera (1:200; Amersham, USA) for 1 h and mounted with DAPI (Vector, Laboratories, CA, USA).

To determine the effect of transduced Tat-HSP22 protein against neuronal cell survival and delayed neuronal damage in ischemic animal model, we performed CV, GFAP, Iba-1, and F-JB immunohistochemistry as previously described [22, 27, 28].

### Cell counting and statistical analysis

For quantification of immunostaining, we performed a cell count in the hippocampal area as described in a previous study [22, 27, 28]. All data are presented as the mean ± SEM. The different between the groups were analyzed using one-way ANOVA with Bonferroni’s test. A value of *P* < 0.05 was considered statistically significant different.

## Abbreviations

Ab: Antibody
BBB: Blood-brain barrier
CV: Cresyl violet
DAPI: 4′,6-diamidino-2-phenylindole
DCF-DA: 2′7′-dichlorofluorescein diacetate
ECL: Enhanced chemiluminescence
F-JB: Fluoro-Jade B
GFAP: Glial fibrillary acidic protein
H_2_O_2_: Hydrogen peroxide
HSP22: Heat shock protein 22
Iba-1: Ionized calcium-binding adapter molecule 1
IPTG: Isopropyl-β-D-thiogalactopyranoside
NeuN: Neuronal neclei
PBS: Phosphate-buffered saline
PTD: Protein transduction domain
ROS: Reactive oxygen species
